# Unraveling the Molecular Mechanism of Selective Antimicrobial Activity of 2(5*H*)-Furanone Derivative against *Staphylococcus aureus*

**DOI:** 10.3390/ijms20030694

**Published:** 2019-02-06

**Authors:** Irshad S. Sharafutdinov, Anna S. Pavlova, Farida S. Akhatova, Alsu M. Khabibrakhmanova, Elvira V. Rozhina, Yulia J. Romanova, Rawil Fakhrullin, Olga A. Lodochnikova, Almira R. Kurbangalieva, Mikhail I. Bogachev, Airat R. Kayumov

**Affiliations:** 1 Laboratory of Molecular Genetics of Microorganisms, Institute of Fundamental Medicine and Biology, Kazan Federal University, 18 Kremlyovskaya street, Kazan 420008, Republic of Tatarstan, Russia; anbio96@gmail.com; 2 Laboratory of Bionanotechnology, Institute of Fundamental Medicine and Biology, Kazan Federal University, Kreml uramı 18, Kazan 420008, Republic of Tatarstan, Russia; farida125@mail.ru (F.S.A.); rozhinaelvira@gmail.com (E.V.R.); kazanbio@gmail.com (R.F.); 3 Biofunctional Chemistry Laboratory, Alexander Butlerov Institute of Chemistry, Kazan Federal University, 18 Kremlyovskaya street, Kazan 420008, Republic of Tatarstan, Russia; alsu-khabibrakhmanova@mail.ru (A.M.K.); lod_olga@mail.ru (O.A.L.); almira99@mail.ru (A.R.K.); 4 Interdisciplinary Centre for Proteomic Research, Institute of Fundamental Medicine and Biology, Kazan Federal University, 18 Kremlyovskaya street, Kazan 420008, Republic of Tatarstan, Russia; magnolina@list.ru; 5 Arbuzov Institute of Organic and Physical Chemistry, FRC Kazan Scientific Center of RAS, 8 Arbuzov Street, Kazan 420088, Republic of Tatarstan, Russia; 6 Biomedical Engineering Research Centre, St. Petersburg Electrotechnical University, St. Petersburg 197022, Russia; rogex@yandex.com

**Keywords:** 2(5*H*)-furanones, biofilms, drug discovery, ROS, Staphylococci

## Abstract

*Staphylococcus aureus* causes various infectious diseases, from skin impetigo to life-threatening bacteremia and sepsis, thus appearing an important target for antimicrobial therapeutics. In turn, the rapid development of antibiotic resistance and biofilm formation makes it extremely robust against treatment. Here, we unravel the molecular mechanism of the antimicrobial activity of the recently unveiled **F105** consisting of three pharmacophores: chlorinated 2(5*H*)-furanone, sulfone, and *l*-menthol moieties. **F105** demonstrates highly selective activity against Gram-positive bacteria and biofilm-embedded *S. aureus* and exhibits low risk of resistance development. We show explicitly that the fluorescent analogue of **F105** rapidly penetrates into Gram-positive bacteria independently of their cell integrity and viability and accumulates there. By contrast, Gram-negative bacteria remain impermeable and, therefore, insusceptible to **F105**. Apparently, in bacterial cells, **F105** induces reactive oxygen species (ROS) formation and nonspecifically interacts with a number of proteins, including ROS-utilizing ones. Using native and 2D PAGE, we confirm that **F105** changes the charge of some proteins by either oxidation or direct interaction with them. Therefore, it seems justified to conclude that being simultaneously a ROS inducer and damaging proteins responsible for ROS utilization, **F105** impairs the cellular anti-ROS defense representing a prospective ROS-inducing antibacterial agent.

## 1. Introduction

While embedded into biofilm, bacteria appear to be strongly protected from various outer stresses such as antimicrobials and the immune system of the host [1,2]. To facilitate the treatment of biofilm-embedded pathogens, the combination of antibiotics with various agents repressing or destroying the biofilm has been suggested as a promising strategy [3]. Among various low molecular weight compounds being potential biofilm-repressing agents (for a detailed review see [3,4]) the derivatives of 2(5*H*)-furanone have been intensively investigated [5]. These compounds were originally described as a natural tool of red algae *Delisea pulchra*, repressing its biofouling [6,7]. It has been shown that various furanones can either be produced naturally by a variety of microorganisms and plants or synthesized chemically [8,9,10]. In cells, furanones participate in intra- and inter-species signaling and communication, and act as attractants, pheromones, and antimicrobials [11]. The discovery of their biofilm suppression activity [6,7] gave rise to intensive investigation of these compounds as biofilm-preventing agents [5,6,12,13,14]. 

The biofilm-preventing activity of 2(5*H*)-furanone derivatives against Gram-negative bacteria is implemented through interference with molecules of autoinducer-1 (AI-1) [15,16,17] and autoinducer-2 (AI-2) [6,7], in particular by competitive replacement of *N*-3-(oxohexanoyl)-L-homoserine lactone (AHL) from its receptor LuxR [6,15]. The molecular targets of 2(5*H*)-furanone derivatives in Gram-positive bacteria remain unknown. While no AHL-signaling is present in Gram-positive bacteria and inter-cellular communication in these microorganisms is governed by short peptides, various synthetic furanones suppress biofilm formation by Gram-positive *S. epidermidis* and *B. subtilis*, while the exact molecular targets are still unknown [7,13,14,18]. Thus, the response of *B. subtilis* to furanones has been reported to be QS-independent [7,18]. By contrast, some data support the idea that furanones somehow affect the QS-processes in *Staphylococci*. Thus, Zang et al. [19] have demonstrated that brominated derivative of furanone covalently binds and, thus, inactivates *S*-ribosylhomocysteine lyase LuxS, the enzyme of the autoinducer-2 synthesis pathway. Kuehl et al. [20] have also shown that LuxS could be a target for furanones in *Staphylococci*. On the one hand, the downregulation of *luxS* expression by subinhibitory concentrations of furanone was observed. On the other hand, in the *luxS* mutant that appeared more active in biofilm formation than its wildtype counterpart, no changes in the biofilm could be observed in the presence of furanones. Lönn-Stensrudet et al. reported that the bioluminescence of *V. harveyi* BB170 could be induced by *S. epidermidis* supernatant, which indicates that Staphylococci use AI-2 for communication. Of note, the induction of bioluminescence was not observed in the presence of furanones, and the biofilm-preventing activity of furanones against *S. epidermidis* was suppressed in the presence of (*S*)-4,5-dihydroxy-2,3-pentanedione (DPD), which is the precursor of AI-2. These data clearly demonstrate that bacterial quorum sensing seems to be one of the apparent targets of furanones also in Gram-positive bacteria. Besides their biofilm-preventing activity, some 2(5*H*)-furanone derivatives also exhibit antibacterial activities [18,21,22], while the molecular targets of these compounds still remain unknown.

Here we show that 3-chloro-5(*S*)-[(1*R*,2*S*,5*R*)-2-isopropyl-5-methylcyclohexyloxy]-4-[4-methylphenylsulfonyl]-2(5*H*)-furanone (**F105**), consisting of three pharmacophores including chlorinated 2(5*H*)-furanone, sulfonyl, and *l*-menthol moieties, exhibits antibacterial activity against biofilm embedded *S. aureus* by producing reactive oxygen species (ROS) and consequent damage of intracellular proteins.

## 2. Results

### 2.1. Antimicrobial Spectrum of ***F105***

In the original research, the antimicrobial activity of 2(5*H*)-furanone **F105** was reported only for *S. aureus*, while the compounds were active against both methicillin-sensitive and -resistant strains [21]. Extended assays of **F105** revealed its highly specific antibacterial activity against Gram-positive bacteria. In particular, **F105** at 8–16 μg/mL repressed the growth and at 32 μg/mL killed 99.9% of the cells of *S. aureus*, *S. epidermidis*, *B. cereus*, *B. subtilis*, and *M. luteus*, while the growth of Gram-negative *K. pneumoniae*, *S. marcescens*, *P. aeruginosa*, and *E. coli* remained unaffected, even at 128 μg/mL of the compound (Table 1). *S. aureus*, *B. cereus*, *S. marcescens*, and *K. pneumonia* have been chosen for further investigations as representative nosocomial pathogenic bacteria with different shapes and cell wall structures.

### 2.2. Synthesis of Fluorescent Compound ***6*** (***F145***)

**Figure 1:**
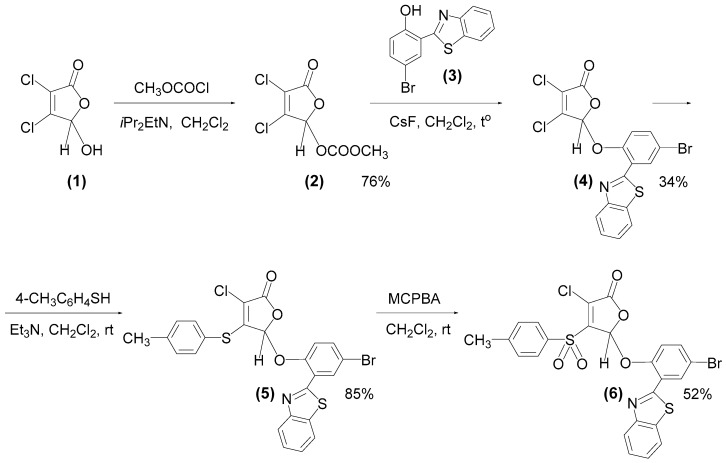
Synthesis of a fluorescent derivative of 2(5*H*)-furanone **6** (**F145**).

Next, to examine whether the observed inefficiency of **F105** against Gram-negative bacteria is due to the absence of molecular targets or their impermeability to furanone, the **F105** analogue possessing a fluorescent moiety and designed as **F145** was synthesized (Figure 1). 5-[2-(Benzothiazol-2-yl)-4-bromophenoxy]-3-chloro-4-[(4-methylphenyl)sulfonyl]-2(5*H*)-furanone (**6)**, studied compound **F145**) was prepared from commercially available mucochloric acid **1** as shown in Figure 1. The residue of 2-(benzothiazol-2-yl)-4-bromophenol (**3**) was chosen as the fluorophore and was synthesized from the reaction of 5-bromosalicylaldehyde with 2-aminothiophenol according to the known procedure [23]. Mucochloric acid methyl carbonate **2** was obtained as described previously [24]. The heating of compound **2** with phenol **3** in the presence of CsF in dichloromethane and subsequent purification of the reaction mixture by silica gel column chromatography led to isolation of the fluorophore-labelled furanone **4**.

At the next stage, a fragment of *p*-thiocresol was introduced into the molecule of compound **4** under basic catalysis. It is well-known that in the presence of triethylamine, reactions of thiols with mucochloric acid **1** and its 5-alkoxy derivatives proceed with the regioselective substitution of the chlorine atom in the fourth position of the lactone ring [25,26,27]. Thiolation reaction was performed in methylene chloride at room temperature with the equimolar ratio of the reactants and Et_3_N (Figure 1). As a result, we isolated the novel thioether **5** with 85% yield. To obtain the corresponding sulfonyl derivative, compound **5** was exposed to *m*-chloroperbenzoic acid (MCPBA). The reaction was carried out in dichloromethane at room temperature using an excess of oxidant resulting in the novel fluorescent sulfone of 2(5*H*)-furanone series **6** (**F145**). Structures of all synthesized compounds were elucidated using ^1^H, ^13^С{^1^H} NMR spectroscopy and HRMS (Figures S1–S4).

**Figure 2:**
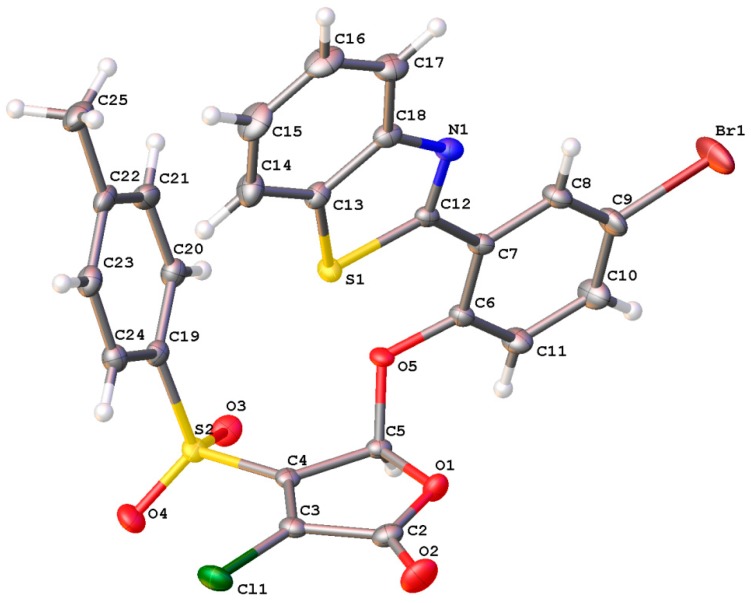
Molecular structure of compound **6** (**F145**) in the crystal.

In addition, the molecular and crystal structure of furanone **6** was characterized using single crystal X-ray diffraction (Figure 2). According to the X-ray data, compound **6** is represented by the chloroform solvate in the examined crystal in the main compound/solvent ratio = 2:1. The chloroform molecule in the crystal is in the special position in the center of symmetry. The overall conformation of the molecule **6** can be described as *cisoid*—aromatic substituents are situated on one side of the plane of the heterocyclic fragment.

### 2.3. Penetration of ***F105*** Analogue into Bacterial Cells

**Figure 3:**
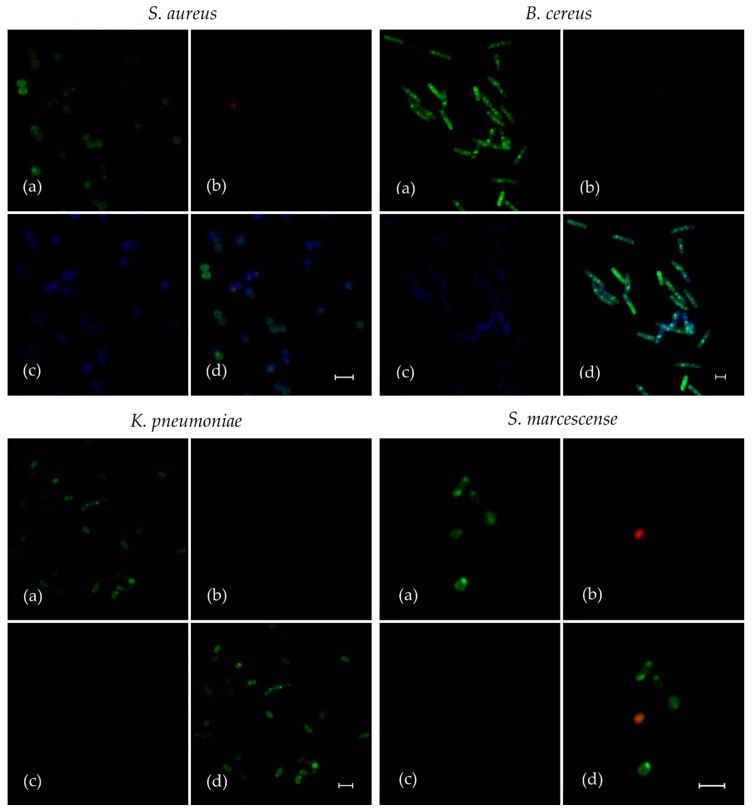
The analysis of fluorescent **F105** analogue (**F145**) penetration into planktonic bacterial cells. Gram-positive (*S. aureus* and *B. cereus*) and Gram-negative (*S. marcescens* and *K. pneumonia*) bacteria were grown for 24 h with agitation in MH broth, then washed and resuspended in PBS. **F145** was added until the final concentration of 10 μg/mL (close to MIC of **F105** for Gram-positive bacteria), and incubation was followed for the next 15 min. Then, the cells were live/dead stained with DioC_6_/PI (additional 15 min) and analyzed with confocal laser scanning microscopy. Images show the fluorescence of DioC_6_ (**a**), PI (**b**), **F145** (**c**) and the combination of all channels (**d**). The scale bars indicate 2.5 µm.

The substitution of *l*-menthol by fluorescent moiety drastically reduced the antibacterial activity of **F145**. Its MICs for all Gram-positive bacteria became 128 mg/L and higher, confirming the strong requirement of *l*-menthol pharmacophore for activity, as was shown earlier [18,21]. Nevertheless, the planktonic bacteria were treated with **F145** for 30 min and its penetration into the cells was assessed with CLSM (Figure 3). Cells were presumed as viable (Figure 3a) due to the fact that only single occurrences of red-stained (dead) cells were observed (Figure 3b). In accordance with antibiotic susceptibility test results (Table 1), the blue fluorescence of **F145** was observed only in *S. aureus* and *B. cereus*. Moreover, as long as DioC_6_ dye stains bacterial cell membrane and accumulates in the cell edge, **F145** fluorescence is distributed evenly in the cells (Figure 3c), which strongly suggests that **F145** penetrates into the cell and accumulates there. Notably, as can be observed in spatial overlap between three fluorescent labels (Figure 3d), **F145** was detected in both alive and dead *S. aureus* and *B. cereus* cells. These data clearly indicate that **F145** easily permeates the cell membrane *S. aureus* and *B. cereus*, but *S. marcescens* and *K. pneumonia* seem to remain impermeable for the furanone.

### 2.4. Penetration of **F105** Analogue into S. aureus Biofilm

**Figure 4:**
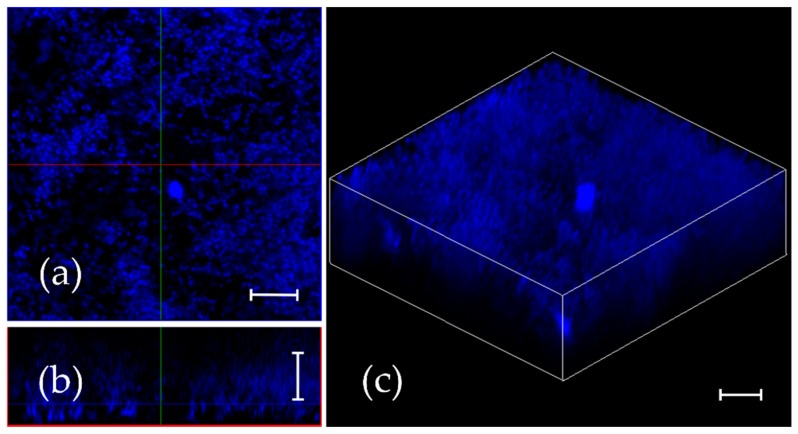
**F145** diffusion into the mature *S. aureus* biofilm. The 24 h old biofilm was treated with **F145** for 1 h and analyzed with confocal laser scanning microscopy using a single-channel mode. (**a**) X; Y orientation of the biofilm; (**b**) Z-stack of the biofilm; (**c**) 3D-model of the biofilm. The scale bars indicate 10 μm.

Earlier we have shown that **F105** efficiently eradicates the mature biofilms of *S. aureus* providing complete death of biofilm-embedded *S. aureus* at 4 × MBC [21]. To investigate the diffusion ability of 2(5*H*)-furanone derivative into the biofilm matrix, *S. aureus* cells were grown in MH broth for 24 h under static conditions to obtain a mature biofilm, and **F145** was added until final concentration of 10 μg/mL. After 1 h of incubation, the biofilm was analyzed with CLSM. The fluorescence of **F145** could be observed throughout all layers of the *S. aureus* biofilm of approximately 20 μm thickness, indicating rapid penetration of the furanone through the biofilm matrix (Figure 4). Moreover, fluorescence signal demonstrated homogeneous distribution of **F145** through the biofilm (Figure 4b) with the predominant accumulation within individual bacterial cells including bottom layers. 

### 2.5. Reactive Oxygen Species (ROS) Induction

**Figure 5:**
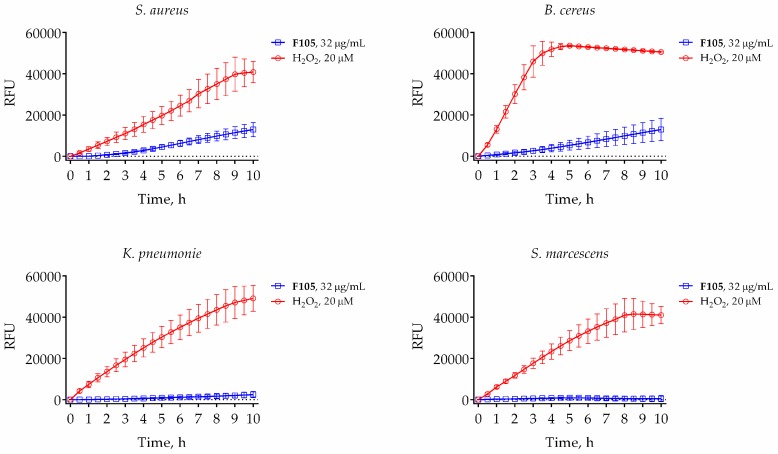
Dynamics of reactive oxygen species (ROS) production in bacteria induced by 20 μM of hydrogen peroxide (red line) or 32 μg/mL of **F105** (blue line). Black line stands for untreated control cells. Bacterial cells were grown for 18 h, harvested, and washed with PBS. Cells were re-suspended until the final density of 10^5^ CFU/mL in PBS supplemented with 2′,7′-dichlorofluorescin diacetate (DCFDA) (5 μM). After 30 min of pre-incubation at 25 °C, 32 μg/mL **F105** or 20 μM H_2_O_2_ were added and the fluorescence was measured for 9 h with 5-min time intervals.

Considering the fact that **F105** contains a chemically active sulfonyl group, we supposed that it might behave as oxidizing agent and probably induce ROS formation. To check this assumption in vivo, we used a cell-permeable 2′,7′-dichlorofluorescin diacetate (DCFDA), which provides sensitive and rapid quantitation of ROS in response to oxidative metabolism. As could be seen from Figure 5, **F105** at the concentration of 32 μg/mL (corresponding to its MBC) led to the significant induction of fluorescence only in *S. aureus* and *B. cereus*. In *S. marcescens* and *K. pneumonia* the fluorescence was comparable with untreated cells (Figure 5). These data clearly indicate that **F105** causes oxidative stress, which probably leads to oxidation of proteins and consequent cell death.

### 2.6. Effect of ***F105*** on Membrane Potential

**Figure 6:**
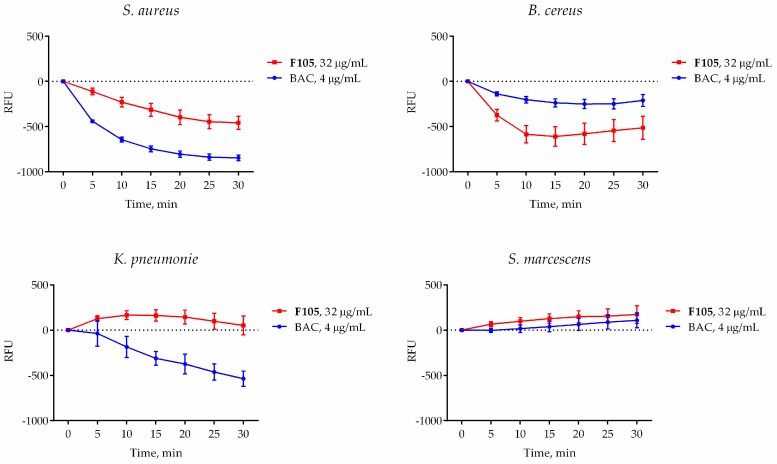
The effect of **F105** (blue line) and benzalkonium chloride (red line) on the membrane potential of bacterial cells. Bacteria were grown for 18 h, harvested, and washed with PBS. Cells were re-suspended until the final density of 10^5^ CFU/mL in PBS supplemented with DioC_2_ (10 μM). After 30 min preincubation at 25 °C, **F105** or BAC were added until final concentrations of 4 or 32 μg/mL, respectively (corresponding to their respective MBCs), and the fluorescence was measured for 30 min with 5-min intervals. Black dashed line indicates the same data for the untreated control cells.

Cell membrane damage, a well-known mechanism of antimicrobial activity exhibited by both ROS and various antimicrobials including quaternary ammonium salts and the lipopeptide antibiotic daptomycin [28], is followed by a drop of the membrane potential. Since ROS production could result in damage of both the intracellular proteins and the membrane, the changes in membrane potential of bacterial cells in response to **F105** treatment was measured by detection of DioC_2_ fluorescence. As a control, bacteria were treated with benzalkonium chloride (BAC), a quaternary ammonium compound which destroys bacterial cells by disrupting its cell membrane. In cells with an intact electrical potential gradient, the green fluorescence of DioC_2_ could be observed. As expected, 4 μg/mL of BAC (corresponding to its MBC for *S. aureus*) led to significant decrease of fluorescence in all treated cells, although with different ranges, suggesting that the membrane potential decreases due to membrane integrity damage (Figure 6). In the presence of **F105**, the fluorescence level in *S. aureus* and *B. cereus* also drastically decreased in comparison with untreated control cells. In marked contrast, in *S. marcescens* and *K. pneumonia* the fluorescence remained almost unchanged in the presence of **F105**, suggesting that their membranes remained unaffected and intact (Figure 6). These data further confirm the specificity of **F105** activity against Gram-positive bacteria.

### 2.7. Effect of ***F105*** on S. aureus Cell Integrity

Additionally, the integrity of *S. aureus* cells after **F105** treatment was analyzed using AFM. *S. aureus* cells were treated for 18 h with 1 × MBC of **F105** (32 μg/mL) and then analyzed with atomic force microscopy. As a control, cells were treated with BAC. 

**Figure 7:**
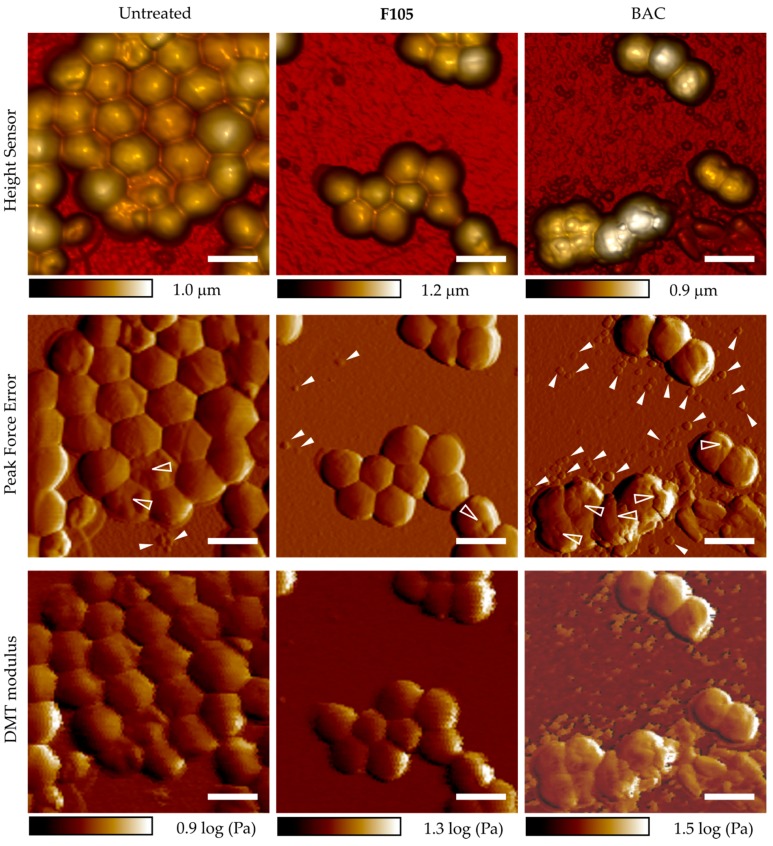
Atomic force microscopy (peak force tapping mode) images of *S. aureus* planktonic cells after 18 h incubation with 4 μg/mL of BAC or 32 μg/mL of **F105** (in correspondence with their respective MBCs). Scale bar is 2 µm. Cell wall damage is shown with transparent arrows; cell debris particles and organic substances are shown with white arrows).

The exposition of cells to BAC at 1 × MBC (4 μg/mL) led to visible cell wall damage and leakage of cell content (Figure 7): cells were characterized with roughened or even split surfaces (shown with transparent arrows), and cell debris particles/organic substances were observed around the cells (shown with a white arrows). At the same time, **F105** at bactericidal concentration (32 μg/mL) led to the same rate of CFU decrease (Table 2) but did not lead to any visible destruction of the surfaces of the treated *S. aureus* cells (Figure 7). Furthermore, no leakage of the intracellular content and cell debris could be observed, which suggests that the cell wall remained intact, in contrast to the BAC treatment (Figure 7). These data suggest that **F105** most likely do not directly target the membrane or the cell wall of bacteria.

### 2.8. ***F105*** Nonspecifically Interacts with a Number of Intracellular Proteins of S. aureus

Only a few investigations have reported about the molecular targets of 2(5*H*)-furanone derivatives in Gram-positive bacteria [13,18,19]. To characterize the mechanism of **F105** antibacterial activity, we investigated changes in the *S. aureus* proteome induced by exposition to **F105**. For that reason, bacterial cells were grown with shaking for 24 h at 37 °C in the presence of a sub-lethal concentration (MIC) of **F105**, and then harvested. Crude extracts from treated and non-treated cells were prepared, stained with Cy5 and Cy3 respectively, and separated by two-dimensional electrophoresis (Figure S5). The observed domination of green spots on a gel suggested significant down-regulation of many proteins in cells when grown in the presence of sub-lethal **F105** concentrations (8 μg/mL). The green-stained spots (proteins which are present only in control cells) and the red-stained spots (proteins expressed in response to **F105** treatment) were excised and identified with LC-MS. Among down-regulated proteins, 13 proteins were identified in each of the three independent experiments (Table S2). The identified proteins are known to be involved in many different cellular processes, from energy metabolism and DNA synthesis to protein folding and cell stress response, and they do not belong to any particular molecular pathway. Similarly, the up-regulated proteins (Table S3) also did not show belonging to any single group, supposing non-specific interaction of **F105** with cellular proteins. 

The negatively charged sulfonyl group of **F105** was supposed to interact with positively charged amino acids of proteins. In this case, one could expect the changes in both charge and conformation of the protein and consequent changes in its electrophoretic mobility under native conditions. To test whether **F105** is able to interact with proteins, the crude extract of *S. aureus* was treated with **F105** (64 μg/mL) for 1 h at 37 °C and the electrophoretic mobilities of proteins from treated and non-treated extracts were compared using 2D-electrophoresis (Figure S6). Again, a number of green and red spots were detected, suggesting that **F105** interacts in vitro with some proteins leading to the changes in their charges and consequent in-gel mobility shift during the first separation by the isoelectric point (pI) value of the protein. The protein spots stained in green and red were also isolated and identified with LC-MS (Table S4). 

Interestingly, five proteins including enolase, molecular chaperone GroEL, inosine-5-monophosphate dehydrogenase, thioredoxin reductase, and branched-chain alpha-keto acid dehydrogenase were identified also in vivo (Table S2), suggesting that they preferentially interact with **F105**. 

**Figure 8:**
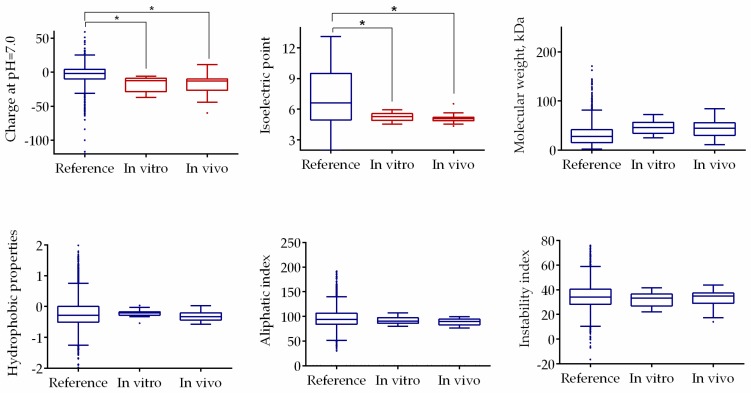
Comparison of physical and chemical properties of the putative substrates for the interaction with **F105** with translated *S. aureus* proteome. Asterisks denote statistically significant differences of medians (* *p* < 0.05).

In an attempt to characterize the putative substrates for **F105,** some characteristics of proteins with changed mobility identified both in vivo and in vitro were predicted in silico and compared with the translated proteome of *S. aureus* (Figure 8). The statistical analysis revealed that typical molecular weights, hydrophobicity, aliphatic, and instability indexes of the proteins identified as putative substrates for **F105** did not have a statistically significant difference from those characteristics calculated for translated proteome. By contrast, these proteins had acidic pI values of around 5.2 and slightly negative charge under physiological pH, which is considerably lower than typical values of whole proteome. This data suggests that **F105** might preferentially interact with acidic, negatively charged proteins. 

**Figure 9:**
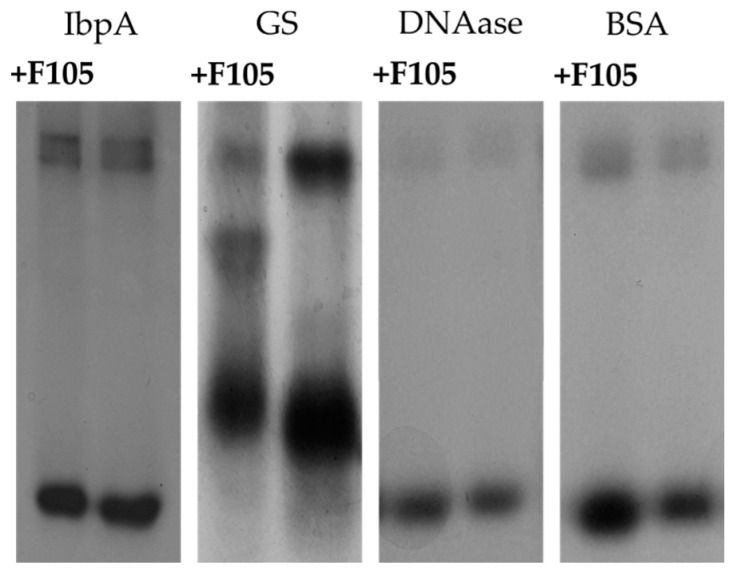
The electrophoretic separation of non-treated and **F105**-treated proteins under native conditions.

To check the assumption that **F105** could interact with proteins, a number of proteins with a pI lower than 7 and negative charge were treated with **F105** and separated with native PAGE in comparison with non-treated samples (Figure 9). Significant changes in migration of **F105**-treated small heat-shock protein IbpA from *Acholeplasma laidlawii* (calculated relative mobility change index 0.971 ± 0.015) and glutamine synthetase from *Bacillus subtilis* (calculated relative mobility change index 0.979 ± 0.004) were detected (Table 3). A slight shift in mobility was observed for DNAase and BSA. These data are in agreement with previous observation that **F105** interacts with proteins and apparently damages their structural or physical-chemical properties leading to the consequent cell death.

## 3. Discussion

To date, molecular targets and mechanisms of 2(5*H*)-furanone derivatives activity against Gram-positive bacteria remain unknown. This work attempts to identify the molecular targets for the 2(5*H*)-furanone derivative **F105** [21], which consists of three pharmacophores including chlorinated 2(5*H*)-furanone, sulfonyl, and *l*-menthol moieties, and exhibits both biofilm suppression and antibacterial activity against Gram-positive bacteria (Table 1). 

Firstly, we have shown that the novel synthesized furanone **F145**, the fluorescent analogue of **F105**, quickly penetrates into the cell of Gram-positive bacteria (*S. aureus* and *B. cereus*) and accumulates there, while Gram-negative *S. marcescens* and *K. pneumonia* remain seemingly impermeable for the furanone (Figure 3). Probably, the overall negatively charged **F105** competes with negatively charged lipopolysaccharides of the outer membrane of these Proteobacteria. Interestingly, **F145** was detected in both viable and dead cells of *S. aureus*, leading to the assumption that the furanone penetrates the cell membrane independently of its integrity and cell viability. 

It has been shown previously that the removal of *l*-menthol or sulfonyl moieties completely suppressed the compound activity [18,21]. Since the *l*-menthol was reported to affect the cell membrane [29] by disruption of the interlamellar hydrogen-bonding network at the polar head group region in the model lipid system [30], it was speculated that the *l*-menthol moiety facilitates rapid diffusion of **F105** into the biofilm matrix and probably through the cell membrane being efficient against both planktonic and biofilm-embedded *S. aureus* cells [21]. While **F145** lacks the *l*-menthol moiety (Figure 1), it was also capable of rapidly diffusing into the lower layers of the biofilm (Figure 4), suggesting that *l*-menthol is not responsible for this property. Moreover, while rather uniformly distributed fluorescence of **F145** in the treated *S. aureus* and *B. cereus* cells could be observed (Figure 3b), neither visible damage of cells nor leakage of the intracellular content could be observed using AFM in the **F105**-treated *S. aureus*, in marked contrast to BAC treatment (Figure 7). Taken together, these data suggest that furanones including **F105** most likely do not directly target the membrane and probably interact with soluble intracellular protein(s). 

Next, since the furanone **F105** possesses a sulfonyl pharmacophore which is known to be chemically active as an oxidizing agent, we suggested that the possible induction of ROS formation in the **F105**-treated cells could play a key role. Indeed, the measurements revealed significant increase of DCFDA fluorescence in the **F105**-treated *S. aureus* and *B. cereus* cells (Figure 5), suggesting ROS formation activation. As additional confirmation of this hypothesis, the induction of heme peroxidase in the **F105**-treated cells could be noted (Table S3). Moreover, the observed decrease of membrane potential in the **F105**-treated *S. aureus* and *B. cereus* cells (Figure 6) could be a consequence of the membrane damage by the ROS. In agreement with no visible indication of **F145** penetration into Gram-negative *S. marcescens* and *K. pneumonia*, for these bacteria neither ROS formation nor decrease of the membrane potential in the presence of **F105** could be observed (Figure 5 and Figure 6). 

In recent years, multiple reports indicated rapid development of antibiotic resistance by *S. aureus* [31], which further speeds up in biofilms [2,32]. To identify the intracellular targets of **F105**, we attempted to obtain a resistant strain. Fourteen serial passages of *S. aureus* on **F105**-containing media did not lead to the development of resistant cells (not shown). On the one hand, only a few antibiotics are effective against *S. aureus* persistent biofilms [1,33], therefore, **F105** seems a promising antimicrobial with low risk of resistance development and highly specific efficiency against biofilm-embedded staphylococci. On the other hand, the classical approach for target identification by comparison of the respective genome or proteome of a sensitive and resistant strain was not available. Therefore, to identify the possible mechanism of **F105** activity, *S. aureus* cells were treated with sublethal concentrations of furanone for 24 h and changes in the proteome were later analyzed. As indicated in Tables S2 and S3, the proteins with changed abundance in treated cells could not be unambiguously identified as belonging to any single group, neither using functional nor pathway-based classification (see Figure S5, Tables S2 and S3). As a control, the crude extract of *S. aureus* was treated with **F105** to evaluate the possibility of direct interactions of furanone with proteins (Figure S6). Again, a number of proteins from the treated extract changed their gel mobility (Table S4), most likely because of the charge alterations caused by the interaction with furanone. It should be further noted that five proteins including enolase, molecular chaperone GroEL, inosine-5-monophosphate dehydrogenase, thioredoxin reductase, and branched-chain alpha-keto acid dehydrogenase were identified both in vivo and in vitro, suggesting that the observed proteome changes (Figure S6) were caused by direct interactions with **F105**. As confirmation of this hypothesis, the comparative analysis revealed high identity of various biochemical properties of these potential substrate proteins (Figure 8). Moreover, the electrophoretic mobility of four individual proteins with pI and charge values close to those of the putative **F105** substrates were changed after 1 h of **F105** treatment (Figure 9, Table 3), suggesting that **F105** could interact with them and affect their properties. Notably, the majority of proteins of *S. aureus* affected by **F105** both in vivo and in vitro were enzymes with positively charged amino acids in their active centers (either His, Lys or Arg), which could interact with furanone as shown in [34]. These data together with the fact of Heme peroxidase induction in the **F105**-treated cells (Table S3) strongly support the hypothesis that **F105** induces the reactive oxygen species (ROS) production with apparent consequent oxidation of proteins. Nevertheless, while the mobility of glutamine synthetase was changed after treatment with **F105** (Figure 9), no significant changes in the enzymatic activity could be observed (not shown), suggesting that **F105** does not necessarily bind with the enzyme’s active center but rather damages the protein structure by yet another mechanism.

Finally, among the proteins affected by **F105** both in vivo and in vitro, we identified two molecular chaperons, GroEL and DnaK, responsible for the protein folding under stress conditions and a set of enzymes responsible for the oxidized proteins reduction (Tables S2–S4). Thus, being on the one hand the ROS inducer, on the other hand, **F105** might bind proteins that are responsible for ROS utilization, thereby damaging the cellular anti-ROS defense. Consequent oxidation of other enzymes involved in common metabolism may lead to their irreversible damage and cell death.

To conclude, **F105** might penetrate the cells and induce oxidative stress, making resistance development highly unlikely. Current development of ROS delivery/induction methods that would be free of side effects for the host tissues represent a promising approach for the topical treatment of infections caused by drug-resistant pathogens [32]. Accordingly, we believe that **F105** is capable of intracellular ROS induction activation and is a promising candidate as a ROS inducing antibacterial agent.

## 4. Materials and Methods

### 4.1. Strains and Growth Conditions

Antibacterial activity of **F105** was evaluated on a number of Gram-positive (methicillin sensitive *Staphylococcus aureus subsp. aureus* ATCC^®^29213 and *Bacillus cereus* (clinical isolate)) and Gram-negative (*Serratia marcescens* and *Klebsiella pneumoniae* (clinical isolates)) bacteria. Clinical isolates were obtained from the Kazan Institute of Epidemiology and Microbiology (Kazan, Russia). The bacterial strains were stored in 10% (V/V) glycerol stocks at −80 °C and freshly streaked on blood agar plates (BD Diagnostics, Franklin Lakes, NJ, USA) and grown overnight at 35 °C before use. Fresh colony material was used to adjust an optical density of 0.5 McFarland (equivalent to 10^8^ cells/mL) in 0.9% NaCl solution that was used as a working suspension and inoculated in Muller-Hinton (MH) broth (Sigma-Aldrich, St. Louis, MO, USA) until final concentration of 10^5^ cells/mL. Benzalkonium chloride (Sigma, St. Louis, Mo, USA) was used as a reference antimicrobial. 

### 4.2. Determination of Minimal Inhibitory (MIC), Minimal Bactericidal Concentrations (MBC) and Resistance Development

The minimum inhibitory concentration (MIC) of **F105** was determined by the broth microdilution method in 96-well microtiter plates (Eppendorf) according to the EUCAST rules for antimicrobial susceptibility testing [35]. The concentrations of test compound ranged from 0.25 to 128 μg/mL. The minimal inhibitory concentration was determined as the lowest concentration of antimicrobial for which no visible bacterial growth could be observed after 24 h of incubation. Then, to determine a minimum bactericidal concentration, a culture liquid from wells without visible growth was diluted a thousand-fold using fresh medium and incubated for 24 h growth at 35 °C. MBC was assumed as a concentration where no viable cells were observed [36]. The development of bacterial resistance was tested using the serial passages approach as described in [37] with modifications as described in [38].

### 4.3. Testing of the Resistance Development

The bacterial resistance development was tested by using the method of serial passages as described in [37] with modifications. In brief, 96-well plates were seeded with bacterial cells at different concentrations of **F105** in a liquid medium with following incubation at 37 °C. After 20 h, microorganisms from the last well with a visible growth (with sub-lethal concentration of a compound) were transferred to an antimicrobial-free agar plate. Then, cells from the agar surface were resuspended in a liquid medium and used as inoculum for next seeding into liquid medium with a range of concentrations of antimicrobials. The procedure was repeated to obtain 14 cycles of passages, and MICs of compounds were determined after each one. Then a series of 7 passages on antimicrobial-free agar was done and MICs were again determined.

### 4.4. Synthesis of 2(5H) Furanone Derivatives and General Chemical Experimental Procedures

The synthesis of **F105** was performed as described in [21]. The detailed synthesis of **F145** is given in the Supplementary Material. NMR spectra were measured on a Bruker Avance III 400 spectrometer at 400.17 MHz (^1^H) and 100.62 MHz (^13^C) at 20 °C in CDCl_3_. The chemical shifts (δ) are reported in parts per million (ppm) calibrated on the residual non-deuterated solvent signal. All coupling constants (*J*) are reported in Hertz (Hz). Multiplicities are indicated as: s (singlet), d (doublet), dd (doublet of doublets), m (multiplet). Analytical thin layer chromatography (TLC) was carried out on Sorbfil PTLC-AF-A-UF plates using UV light (254 nm) as the visualizing agent. Silica gel 60 A (Acros Organics, Morristown, NJ, USA, 0.060–0.200 mm) was used for open column chromatography. The melting points were measured on an OptiMelt Stanford Research Systems MPA100 automated melting point apparatus and were not corrected. High-resolution mass spectra (HRMS) were obtained on a Bruker micrOTOF–QIII spectrometer using electron spray ionization (ESI–TOF–MS).

3,4-Dichloro-5-hydroxy-2(5*H*)-furanone (mucochloric acid, 1) (Vekton, Moscow, Russia) was recrystallized from water, mp 127 °C. 4-Methylthiophenol (Alfa Aesar, Ward Hill, MA, USA), 3-chloroperoxybenzoic acid (Acros Organics), and CsF (TCI) were used as received without further purification. All organic solvents were purified and distilled using standard procedures. 3,4-Dichloro-5-methoxycarbonyloxy-2(5*H*)-furanone (2) [24] and 2-(benzothiazol-2-yl)-4-bromophenol (3) [23] were synthesized according to the previously reported methods. NMR and HRMS spectra are given in the Supplementary Material (Figures S1–S4).

### 4.5. Single Crystal X-ray Analysis

The X-ray diffraction data for the single crystal of 6 were collected on a Bruker Smart Apex II CCD diffractometer (ω-scan mode) using graphite monochromated Mo*Kα* (0.71073 Å) radiation at 150 K. The structure was solved by the direct methods using SHELXT–2014/5 [39] and refined by the full-matrix least-squares on *F*^2^ using SHELXL–2017/1 [40]. Calculations were mainly performed using WinGX–2014.1 suite of programs [41]. Non-hydrogen atoms were refined anisotropically. The hydrogen atoms were inserted at the calculated positions and refined as riding atoms.

The crystal data, data collection and structure refinement details for 6 are summarized in Table S1 of the Supplementary Material. The crystallographic data for the investigated crystal 6 have been deposited in the Cambridge Crystallographic Data Centre as supplementary publication CCDC number 1876720. These data can be obtained free of charge via www.ccdc.cam.ac.uk/data_request/cif, by emailing data_request@ccdc.cam.ac.uk, or by contacting The Cambridge Crystallographic Data Centre, 12 Union Road, Cambridge CB2 1EZ, UK; fax: +44 1223 336033.

### 4.6. Confocal Laser Scanning Microscopy

Planktonic *S. aureus* cells were grown for 24 h with agitation in MH broth, then washed and resuspended in equal volume of PBS. Next, **F145** was added until final concentration of 10 μg/mL and incubated for 15 min, with subsequent additional staining with 5 μM DioC_6_ and 20 μM propidium iodide for the next 15 min. *S. aureus* biofilm was pre-grown for 24 h in MH under static conditions and after washing with PBS was stained with 10 μM **F145** for 1 h. Stained *S. aureus* cells and biofilm were analyzed under vital conditions using an inverse confocal laser scanning microscope LSM780 (Carl Zeiss AG, Jena, Germany) at blue (405/410–508 nm), green (488/490–606 nm), and red (543/566–718 nm) channels. An area of approximately 100 µm (X) × 100 µm (Y) was screened in 1 µm Z-intervals (Z-stack). The obtained data were visualized using ZEN 9.0 software (Carl Zeiss AG).

### 4.7. Atomic Force Microscopy

*S. aureus* cells were grown with agitation at 37 °C in MH broth in the presence of **F105** or BAC for 16–20 h. Then, cells were washed three times with pure water and placed on the cover slide with following air-drying. Atomic force microscopy images of *S. aureus* cells were collected using a Dimension Icon Scanning Probe Microscope (Bruker, Billerica, MA, USA) operating in PeakForce Tapping™ mode at ambient conditions. Scan Asyst-Air probes (Bruker) with nominal length 115 μm, tip radius 2 nm, and spring constant 0.4 N/m were used throughout. The images were obtained at 512 line/scan at 0.8–0.9 Hz scan rate. The images were acquired in height (topography), peak force error, DMT-modulus and adhesion channels. The raw AFM imaging data obtained were processed and analyzed using Nanoscope Analysis v.1.7 software (Bruker). 

### 4.8. ROS Detection

The relative amount of intracellular reactive oxygen species was quantified by using a cell-permeable 2′,7′-dichlorofluorescin diacetate (DCFDA), which is de-esterified intracellularly and turns to highly fluorescent 2′,7′-dichlorofluorescein upon oxidation. Bacteria were grown for 18 h with agitation, harvested, and washed with PBS. Cells were resuspended until final density of 10^5^ CFU/mL in PBS supplemented with DCFDA (5 μM). After 30 min pre-incubation at 25 °C, 32 μg/mL **F105** or 20 μM H_2_O_2_ were added, and the fluorescence was measured for 9 h with 5-min intervals using FAM-filter set detection on Bio-Rad CFX96.

### 4.9. Membrane Potential Evaluation

Membrane potential was evaluated by detection of 3,3′-diethyloxacarbocyanine iodide (DioC_2_(3)) fluorescence. Bacteria were grown for 18 h in MH broth with agitation, harvested, and washed with PBS. Cells were resuspended until final density of 10^5^ CFU/mL in PBS supplemented with 3,3′-diethyloxacarbocyanine iodide (DioC_2_(3)) until final concentration of 10 μM. After 30 min preincubation at 25 °C, **F105** or BAC were added, and the fluorescence was measured for 30 min with 5-min intervals using FAM-filter set detection on Bio-Rad CFX96.

### 4.10. Proteomic Assays

The crude extracts of treated and non-treated cells were stained with Cy5 and Cy3 dyes, respectively. Next, the first dimension of two-dimensional gel electrophoresis was performed by using ready IPG gel strips with pH gradient of 4-7 (Bio-Rad); the second dimension was carried out in 15%-polyacrylamide gel by [42]. The protein spots were excised from the gel, subjected to in-gel trypsinolysis, and identified using LC-MS/MS on a maXis Impact™ Mass Spectrometer (Bruker). Polyacrylamide gel electrophoresis under native conditions was performed as described in [42] with IbpA from *Acholeplasma laidlawii* [43], glutamine synthetase (GS) from *Bacillus subtilis* [44], DNAase, bovine serum albumin (BSA) from *Bos taurus*, and bovine liver catalase as model proteins. The Gs activity was measured as described in [44].

### 4.11. Statistical Analysis

The molecular weight, isoelectric point, aliphatic index, charge, hydropathy, and instability index of proteins have been calculated using http://web.expasy.org/compute_pi/ and http://www.camp.bicnirrh.res.in/featcalc/ tools. Next, the distributions of the calculated physical and chemical properties for all proteins interacting with **F105** in vivo and in vitro as well as the entire pattern obtained from the *S. aureus* genome were compared using statistical Kolmogorov-Smirnov test as previously [45].

## Figures and Tables

**Table 1:** Antimicrobial spectrum of **F105**.

	MIC, μg/mL	MBC, μg/mL
*S. aureus*	8	32
*S. epidermidis*	16	32
*B. cereus*	8	32
*B. subtilis*	16	32
*M. luteus*	8	32
*K. pneumoniae*	>128	>128
*S. marcescens*	>128	>128
*P. aeruginosa*	>128	>128
*E. coli*	>128	>128

**Table 2:** CFUs number of *S. aureus* after 18 h treatment.

Control	F105, 32 μg/mL	BAC, 4 μg/mL
7 × 10^9^	2 × 10^5^	5 × 10^5^

**Table 3:** Properties of proteins.

Protein	IbpA	GS	DNAase	BSA
Charge	−2.3	−18.5	−8.1	−9.9
pI	5.7	5.1	5.3	6.2
Relative mobility change index	0.979 ± 0.004	0.971 ± 0.015	1.016 ± 0.005	1.017 ± 0.004

## Supplementary Materials

Supplementary materials can be found at http://www.mdpi.com/1422-0067/20/3/694/s1. Figure S1: ^1^H (a) and ^13^С{^1^H} (b) NMR spectra of compound **4** (СDCl_3_); Figure S2: ^1^H (a) and ^13^С{^1^H} (b) NMR spectra of compound **5** (СDCl_3_); Figure S3: ^1^H (a) and ^13^С{^1^H} (b) NMR spectra of compound **6** (СDCl_3_); Figure S4: Mass spectra of compounds **4**–**6**; Figure S5: 2D electrophoresis of proteome of *S. aureus* cells treated for 24 hours with sub-lethal concentration (8 μg/mL) of **F105** (red spots), and untreated (green spots). Overlay of two subpopulations of proteins represents yellow spot; Figure S6: 2D electrophoresis of *S. aureus* cell crude extracts after 1-hour treatment with **F105** (64 μg/mL) (red spots), and untreated (green spots). Overlay of two subpopulations of proteins represents yellow spot; Table S1: Crystal data, data collection and structure refinement details for **6** (**F145**); Table S2: Intracellular proteins with decreased amount after growth in the presence of **F105** (LC-MS); Table S3: Intracellular proteins with increased amount after growth in the presence of **F105** (LC-MS); Table S4: *S. aureus* proteins with altered mobility after incubation with **F105** in vitro.

## Abbreviations

**F105**	3-chloro-5(*S*)-[(1*R*,2*S*,5*R*)-2-isopropyl-5-methylcyclohexyloxy]-4-[4-methylphenylsulfonyl]-2(5*H*)-furanone
**F145**	5-[2-(Benzothiazol-2-yl)-4-bromophenoxy]-3-chloro-4-[(4-methylphenyl)sulfonyl]-2(5*H*)-furanone
BAC	Benzalkonium chloride
CFU	Colony forming units
RFU	Relative fluorescence unit
DCFDA	2′,7′-dichlorofluorescin diacetate
DioC_2_(3)	3,3′-diethyloxacarbocyanine iodide
DCFDA	2′,7′-dichlorofluorescin diacetate
MBC	Minimal bactericidal concentration
CLSM	Confocal laser scanning microscopy
AFM	Atomic force microscopy
LC-MS	Liquid Chromatography Mass Spectrometry
MCPBA	*m*-Chloroperbenzoic acid
NMR	Nuclear magnetic resonance spectroscopy
HRMS	High-resolution mass spectrometry
TLC	Thin layer chromatography
