# Supplementary material for: Unraveling the Molecular Mechanism of Selective Antimicrobial Activity of 2(5H)-Furanone Derivative against Staphylococcus aureus
Source: Int J Mol Sci. 2019 Feb 6;20(3):694. doi: 10.3390/ijms20030694 (PMC6387044; doi:10.3390/ijms20030694)
Supplement: Supplementary file 1 [file ijms-20-00694-s001.pdf]

Supplementary Material

Unraveling the molecular mechanism of selective antimicrobial activity of 2(5*H*)-furanone derivative against *Staphylococcus aureus*

Irshad S Sharafutdinov, Anna S Pavlova, Farida S Akhatova, Alsu M Khabibrakhmanova, Elvira V Rozhina, Yulia J Romanova, Ravil F Fakhrullin, Olga A Lodochnikova, Almira R Kurbangalieva, Mikhail I Bogachev, Airat R Kayumov

1. Chemistry

1.1. Synthetic procedures

1.1.1. **5-[2-(Benzothiazol-2-yl)-4-bromophenoxy]-3,4-dichloro-2(5*H*)-furanone (4).** To a stirred solution of furanone **2** (0.30 g, 1.3 mmol) in dichloromethane (18 mL) at room temperature was added CsF (0.26 g, 1.7 mmol) under an Ar atmosphere. Then a solution of phenol **3** (0.45 g, 1.5 mmol) in dichloromethane (12 mL) was added. The reaction mixture was heated at reflux for 24 h. After cooling, the reaction mixture was evaporated to dryness under reduced pressure and the obtained yellow residue was purified by silica gel column chromatography using toluene as the eluent. From the first nonpolar fraction, unreacted phenol **3** (0.17 g, 38%) was recovered. The second fraction contained the desired product **4** (0.20 g, 34%) as colorless solid, mp 210–212 °C (decomp.). TLC R_f 0.29 (toluene). IR, ν, cm^{-1}: 1797 (C=O), 1644 (C=$C_{lactone}$), 1490, 1457 (C=C_{arom}). ^{1}H NMR, δ, ppm: 6.47 (s, 1H, H^5), 7.25 (d, *J* = 6.7 Hz, 1H), 7.39–7.48 (m, 1H), 7.49–7.58 (m, 1H), 7.62 (dd, *J* = 8.8, 2.0 Hz, 1H), 7.95 (d, *J* = 8.1 Hz, 1H), 8.12 (d, *J* = 8.1 Hz, 1H), 8.66 (d, *J* = 2.1 Hz, 1H). ^{13}C{^{1}H} NMR, δ, ppm: 98.54 (C^5), 117.54, 117.91, 121.57, 123.53, 125.69, 126.63, 133.23, 134.55, 136.18, 146.52, 152.14, 152.23, 160.28, 162.32 (C^2, C^3, C^4, C_{arom}). HRMS (ESI) *m/z* calcd for $C_{17}H_9BrCl_2NO_3S$ ([M+H]$^+$) 455.8858, found 455.8857.

1.1.2. **5-[2-(Benzothiazol-2-yl)-4-bromophenoxy]-3-chloro-4-[(4-methylphenyl)sulfanyl]-2(5*H*)-furanone (5).** To a solution of furanone **4** (0.14 g, 0.3 mmol) in dichloromethane (10 mL) with intense stirring was added dropwise a solution of 4-methylthiophenol (0.04 g, 0.3 mmol) in dichloromethane (3 mL), and a solution of triethylamine (0.04 mL, 0.3 mmol) in dichloromethane (1 mL). The reaction mixture was stirred at room temperature for 24 h, then washed with water (30 mL × 3), and the aqueous layer was extracted with dichloromethane (30 mL × 3). The combined organic layers were dried over $MgSO_4$, and the solvent was removed under reduced pressure. The obtained solid residue was recrystallized from hexane to afford thioether **5** (0.14 g, 85%) as colorless solid, mp 175 °C. TLC R_f 0.25 (ethyl acetate / petroleum ether, 1:6). IR, ν, cm^{-1}: 1769 (C=O), 1583, 1491, 1456 (C=C_{arom}). ^{1}H NMR, δ, ppm: 2.24 (s, 3H, CH_3), 6.15 (s, 1H, H^5), 6.64, 6.97 (m, AA'BB', $N = {}^3J_{AB} + {}^5J_{AB'}$ = 8.4 Hz, 4H), 7.25 (d, *J* = 7.6 Hz, 1H), 7.40 (dd, *J* = 8.9, 2.5 Hz, 1H), 7.43–7.50 (m, 1H), 7.52–7.60 (m, 1H), 8.01 (d, *J* = 8.1 Hz, 1H), 8.14 (d, *J* = 8.1 Hz, 1H), 8.61 (d, *J* = 2.5 Hz, 1H). ^{13}C{^{1}H} NMR, δ, ppm: 21.32 (CH_3), 97.37 (C^5), 115.74, 116.85, 118.08, 121.20, 121.70, 123.38, 125.24, 125.64, 126.59, 130.63, 132.70, 134.09, 135.17, 136.21, 141.56, 151.95, 152.06, 155.35, 160.56, 163.81 (C^2, C^3, C^4, C_{arom}). HRMS (ESI) *m/z* calcd for $C_{24}H_{16}BrClNO_3S_2$ ([M+H]$^+$) 543.9438, found 543.9438.

1.1.3. **5-[2-(Benzothiazol-2-yl)-4-bromophenoxy]-3-chloro-4-[(4-methylphenyl)sulfonyl]-2(5*H*)-furanone (6).** To a stirred solution of thioether **5** (0.11 g, 0.2 mmol) in dichloromethane (10 mL) was added dropwise a solution of *m*-chloroperoxybenzoic acid (0.09 g, 0.5 mmol) in dichloromethane (6 mL). The mixture was stirred for 7 h at room temperature, the progress of the reaction was monitored by ^{1}H NMR spectroscopy. When the reaction was complete, the mixture was washed first with 6% aqueous sodium bicarbonate and then with water to pH 7. The aqueous layer was extracted with an additional dichloromethane (20 mL × 2), the combined organic layers were dried over calcium chloride, and the solvent was evaporated to dryness. The yellowish solid residue was purified by silica gel column chromatography (ethyl acetate / petroleum ether, 1:6) followed by recrystallization from a mixture hexane / chloroform (5:6) to give sulfone **6** (0.06 g, 52%) as colorless crystals, mp 210–213 °C (decomp.). TLC R_f 0.17 (ethyl acetate / petroleum ether, 1:6). IR, ν, cm^{-1}: 1804 (C=O), 1621 ($C=C_{lactone}$), 1585, 1491, 1458 ($C=C_{arom}$), 1335 (SO_2^{as}); 1158 (SO_2^{s}). ^{1}H NMR, δ, ppm: 2.04 (s, 3H, CH_3), 6.99 (s, 1H, H^5), 7.06, 7.31 (m, AA'BB', $N = {}^3J_{AB} + {}^5J_{AB'}$ = 8.5 Hz, 4H), 7.36–7.44 (m, 1H), 7.47–7.55 (m, 1H), 7.66 (dd, J = 8.8, 2.4 Hz, 1H), 7.72 (d, J = 8.1 Hz, 1H), 7.79 (d, J = 8.2 Hz, 1H), 8.09 (d, J = 8.1 Hz, 1H), 8.56 (d, J = 2.3 Hz, 1H). $^{13}C\{^1H\}$ NMR, δ, ppm: 21.59 (CH_3), 97.82 (C^5), 116.98, 118.05, 121.39, 123.57, 125.70, 126.22, 126.54, 129.09, 130.24, 133.44, 134.69, 134.82, 136.10, 147.60, 150.61, 151.72, 152.05, 157.61, 160.53, 162.17. HRMS (ESI) *m/z* calcd for $C_{24}H_{16}BrClNO_5S_2$ ($[M+H]^+$) 575.9336, found 575.9340.

1.2. ¹H and ¹³C{¹H} NMR spectra

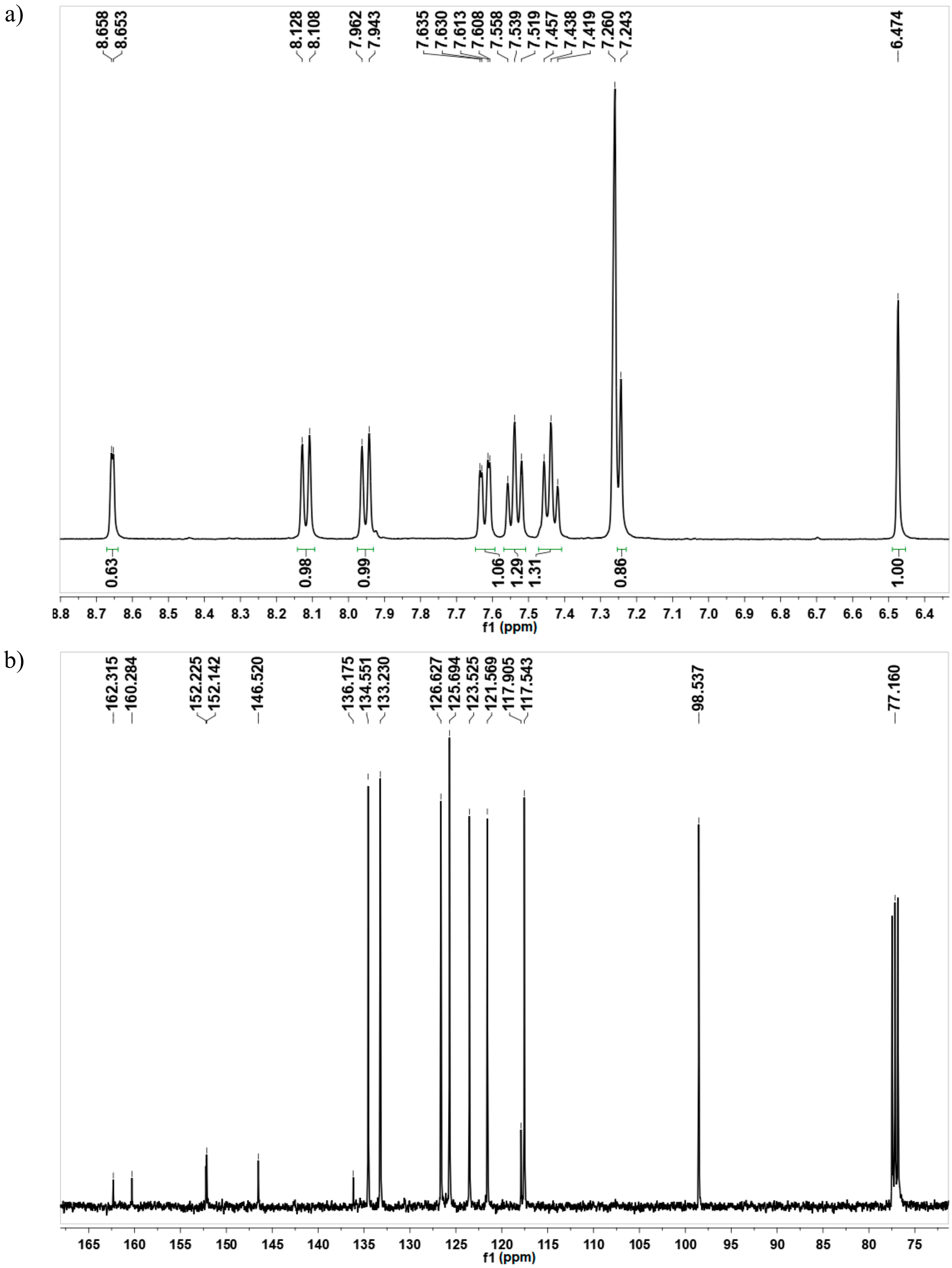

Figure S1. ¹H (a) and ¹³C{¹H} (b) NMR spectra of compound **4** ($CDCl_3$).

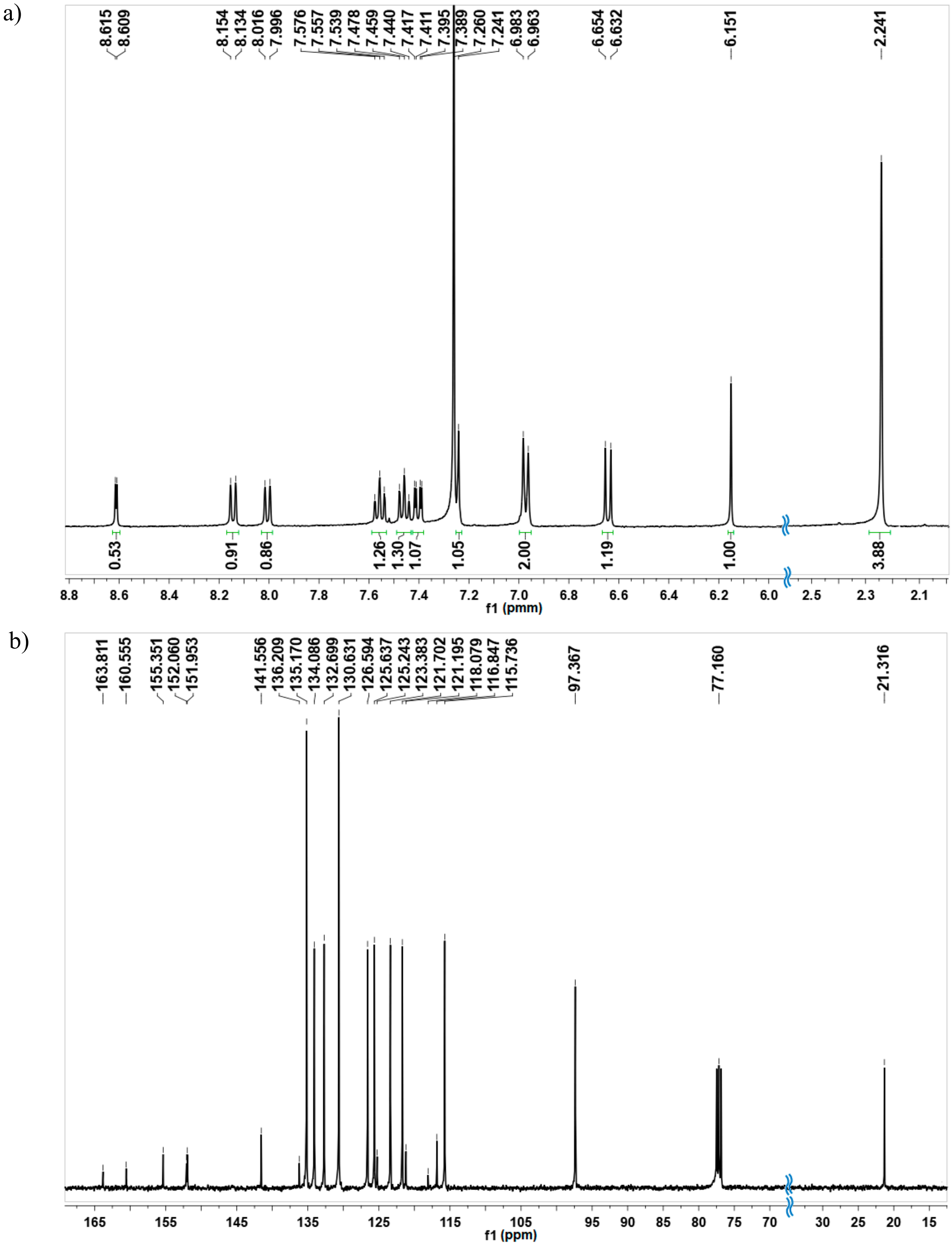

Figure S2. ^1H (a) and $^{13}C\{^1H\}$ (b) NMR spectra of compound **5** ($CDCl_3$).

a)

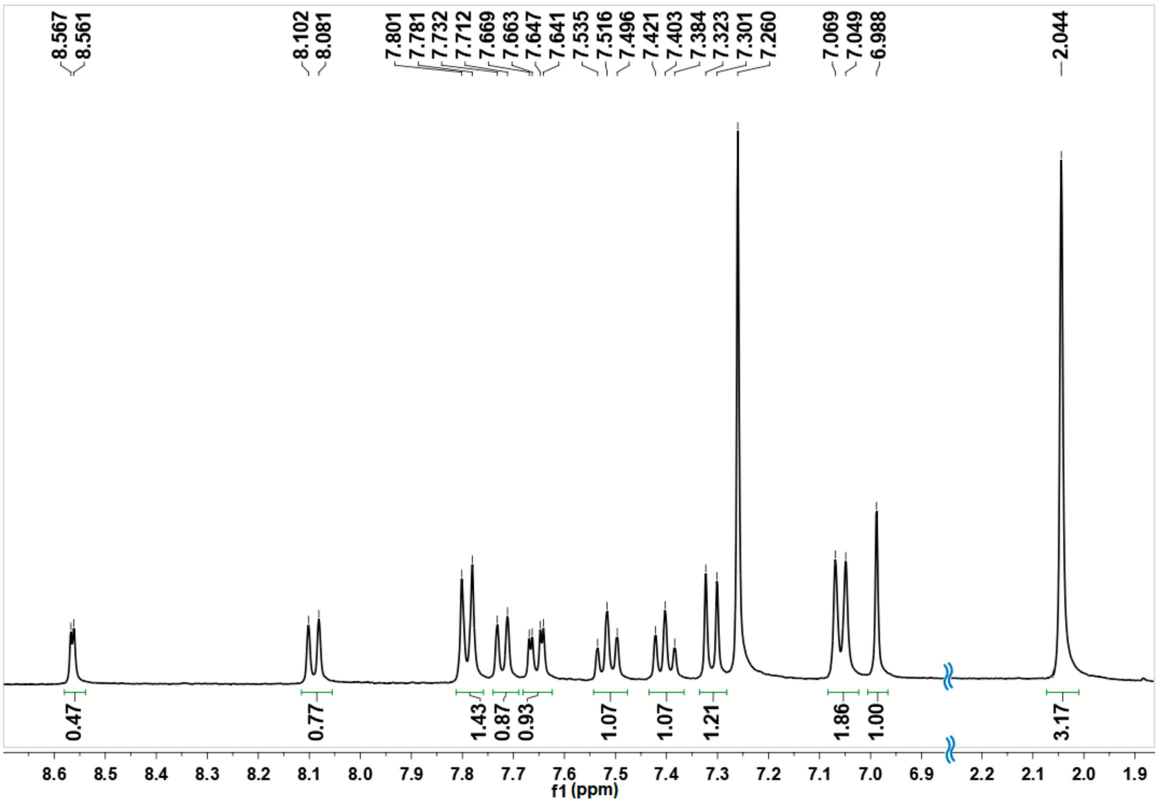

b)

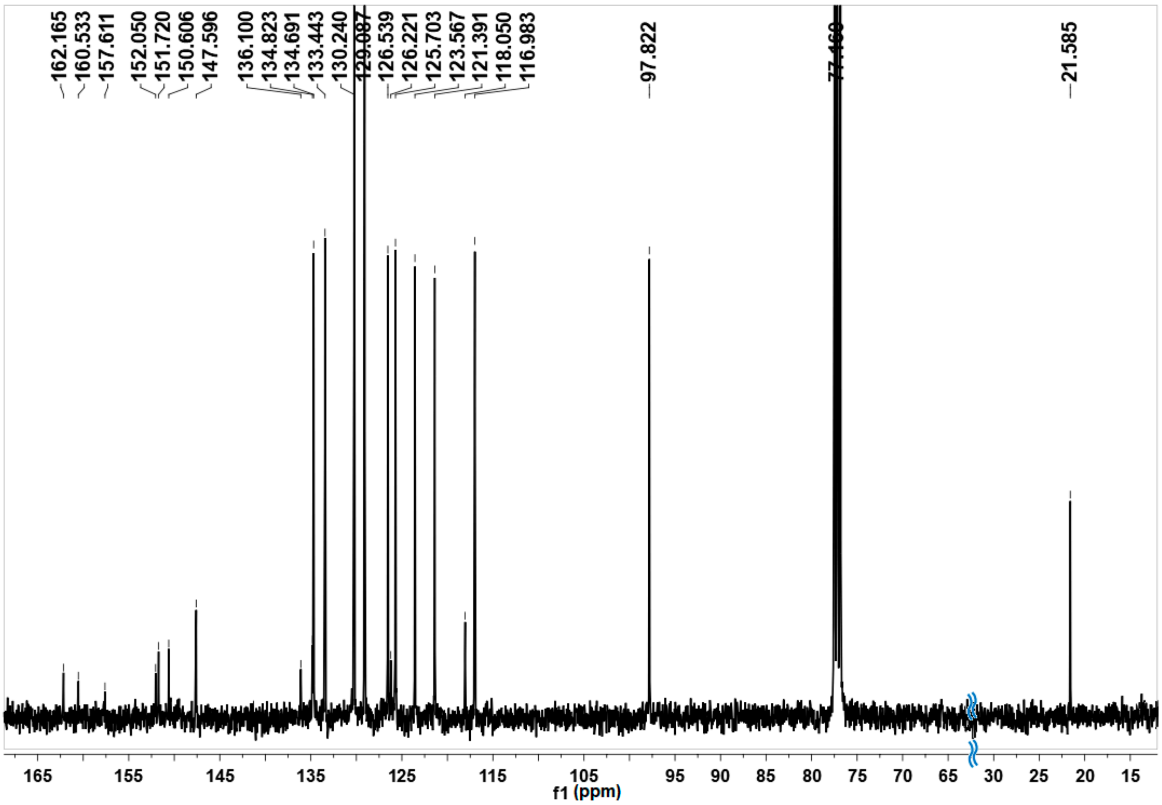

Figure S3. ^{1}H (a) and $^{13}C\{^{1}H\}$ (b) NMR spectra of compound **6** ($CDCl_3$).

1.3. Mass spectra

a)

Acquisition Parameter

Source Type	ESI	Ion Polarity	Positive	Set Nebulizer	3.0 Bar
Focus	Not active			Set Dry Heater	200 • ‹C
Scan Begin	50 m/z	Set Capillary	4500 V	Set Dry Gas	10.0 l/min
Scan End	2000 m/z	Set End Plate Offset	-500 V	Set Divert Valve	Waste

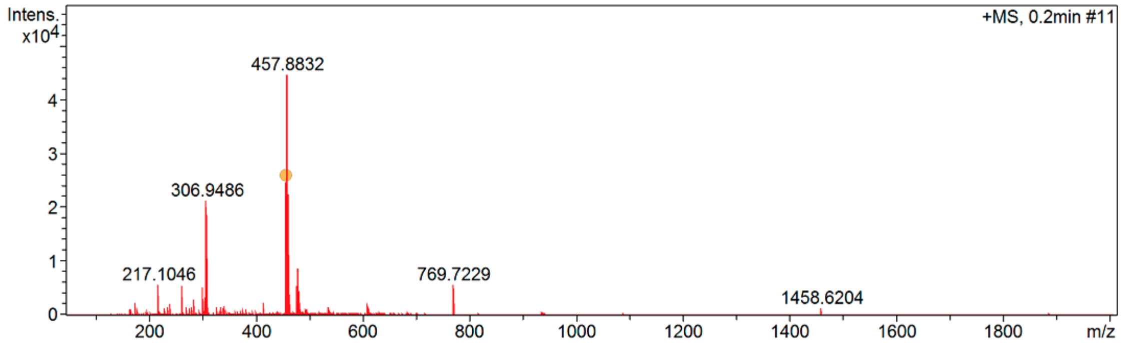

Meas. m/z	#	Ion Formula	m/z	err [ppm]	mSigma	# mSigma	Score	rdb	e•	P Conf	N-Rule
455.8857	1	C17H9BrCl2NO3S	455.8858	0.2	16.8	1	100.00	12.5	even		ok

b)

Acquisition Parameter

Source Type	ESI	Ion Polarity	Positive	Set Nebulizer	3.0 Bar
Focus	Not active			Set Dry Heater	200 • ‹C
Scan Begin	50 m/z	Set Capillary	4500 V	Set Dry Gas	10.0 l/min
Scan End	2000 m/z	Set End Plate Offset	-500 V	Set Divert Valve	Waste

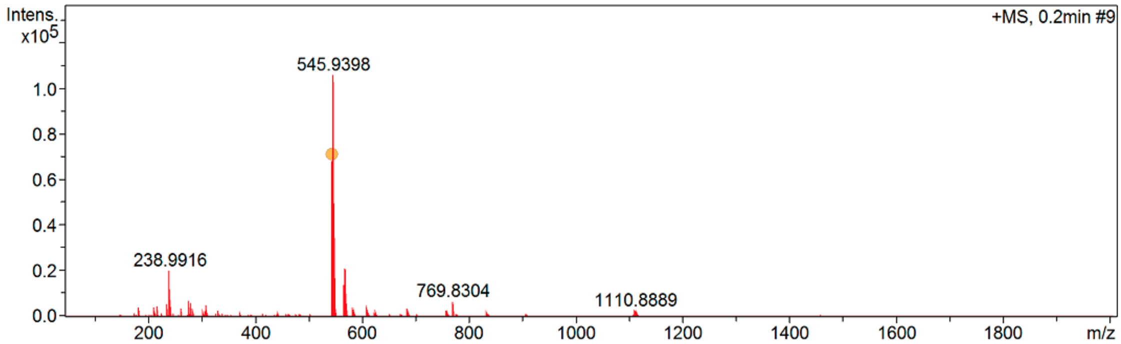

Meas. m/z	#	Ion Formula	m/z	err [ppm]	mSigma	# mSigma	Score	rdb	e•	P Conf	N-Rule
543.9438	1	C24H16BrClNO3S2	543.9438	0.1	25.6	1	100.00	16.5	even		ok

c)

Acquisition Parameter

Source Type	ESI	Ion Polarity	Positive	Set Nebulizer	3.0 Bar
Focus	Not active			Set Dry Heater	200 • ‹C
Scan Begin	50 m/z	Set Capillary	4500 V	Set Dry Gas	10.0 l/min
Scan End	2000 m/z	Set End Plate Offset	-500 V	Set Divert Valve	Waste

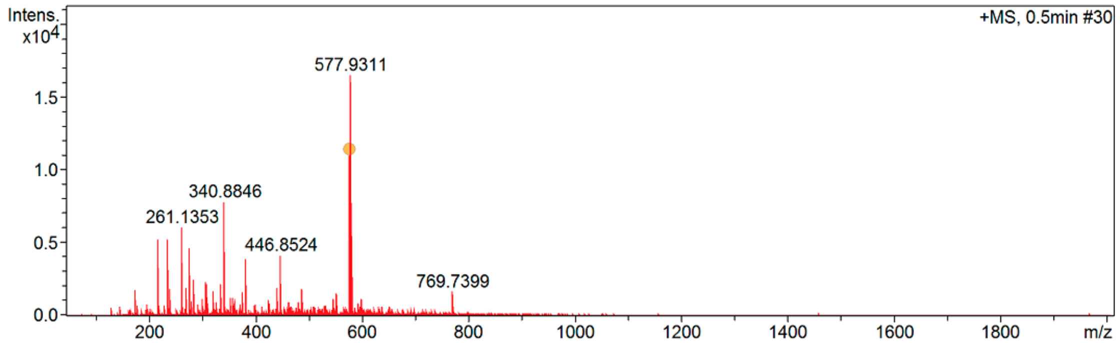

Meas. m/z	#	Ion Formula	m/z	err [ppm]	mSigma	# mSigma	Score	rdb	e•	P Conf	N-Rule
575.9340	1	C24H16BrClNO5S2	575.9336	-0.7	24.5	1	100.00	16.5	even		ok

Figure S4. Mass spectra of compounds **4**–**6**.

1.4. Single crystal X-ray analysis

Table S1. Crystal data, data collection and structure refinement details for **6** (**F145**)

Empirical formula	$C_{24}H_{15}BrClNO_5S_2$ 0.5 $CHCl_3$
Formula weight	636.53
Temperature	150(2) K
Radiation, wavelength	Mo$K\alpha$, 0.71073 Å
Crystal system	Triclinic
Space group	P–1 (No. 2)
Unit cell dimensions	a = 10.6224(10) Å, b = 10.9807(12) Å,<br>c = 11.9831(12) Å, α = 114.055(5)°<br>β = 92.733(5)°, γ = 98.241(5)°
Volume	1254.4(2) $Å^3$
Z and Z'	2 and 1
Calculated density	1.685 g·cm^{-3}
Absorption coefficient	2.110 mm^{-1}
F(000)	638
Θ range for data collection	3.1° to 28.0°
Index ranges	$-14 \leq h \leq 14$<br>$-14 \leq k \leq 14$<br>$-15 \leq l \leq 15$
Reflections collected	6344
Independent reflections	31555 [R(int) = 0.036]
Observed data [$I > 2\sigma(I)$]	5331
Data / parameters	6049 / 348
Goodness-of-fit on F^2	1.06
Final R indices [$I > 2\sigma(I)$]	R_1 = 0.0365, wR_2 = 0.0795
R indices (all data)	R_1 = 0.0436, wR_2 = 0.0832
Largest diff. peak and hole	–2.53 and 1.98 e·$Å^{-3}$

2. Biology

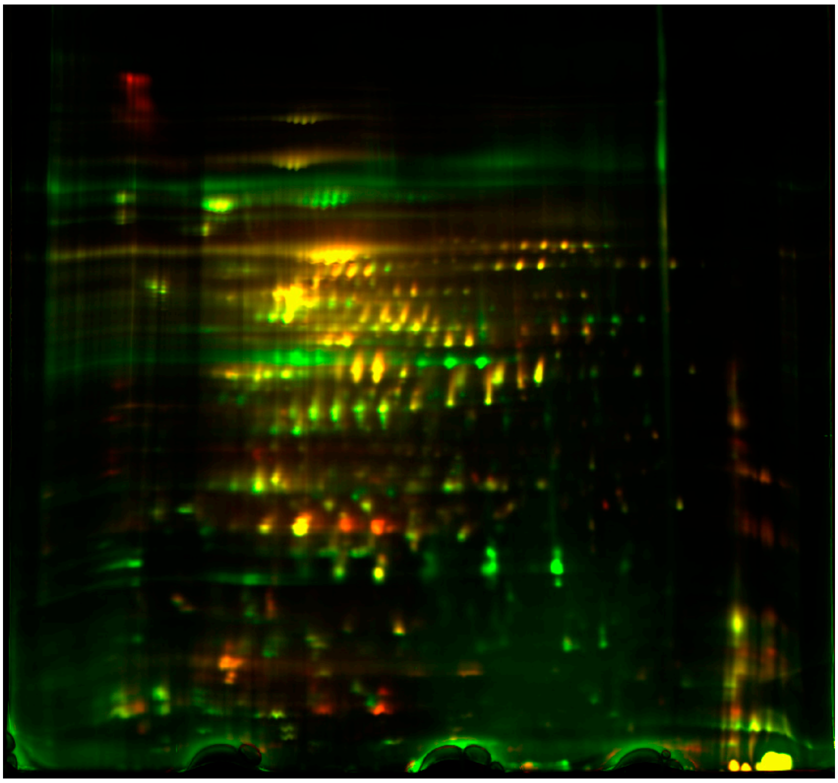

Figure S5. 2D electrophoresis of proteome of *S. aureus* cells treated for 24 hours with sub-lethal concentration (8 µg/ml) of **F105** (red spots), and untreated (green spots). Overlay of two subpopulations of proteins represents yellow spot

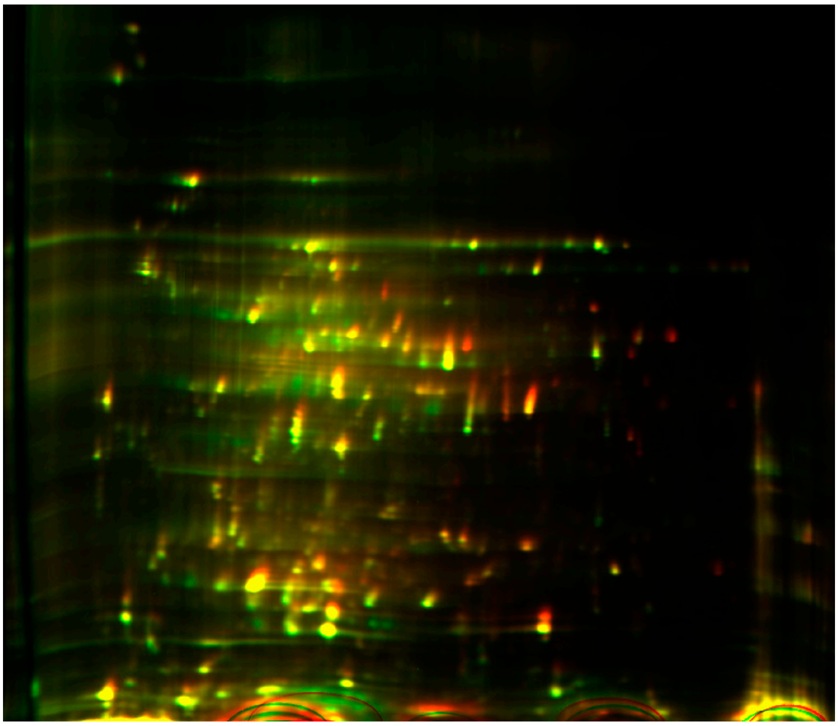

Figure S6. 2D electrophoresis of *S. aureus* cell crude extracts after 1-hour treatment with **F105** (64 µg/ml) (red spots), and untreated (green spots). Overlay of two subpopulations of proteins represents yellow spot

Table S2. Intracellular proteins with decreased amount after growth in the presence of **F105** (LC-MS).

#	Protein	Function
1	DNA-directed RNA polymerase subunit beta~	RNA synthesis
2	Methionine--tRNA ligase	l-methionyl-tRNAMet biosynthesis
3	Molecular chaperone GroEL	folding of many proteins
4	Inosine-5-monophosphate dehydrogenase	biosynthesis of guanine nucleotides
5	Alkaline phosphatase	Dephosphorylation
6	Enolase	glycolysis
7	Branched-chain alpha-keto acid dehydrogenase subunit E2	catalyzes a transfer of the acyl group from the lipoyl moiety to coenzyme A (a stoichiometric cofactor)
8	Thioredoxine reductase	redox signaling, reduction of thioredoxin
9	Glycerophosphodiester phosphodiesterase	glycerophospholipid metabolism (the main component of biological membranes)
10	Fructose-1,6-bisphosphate aldolase	glycolysis
11	30S ribosomal protein S3	binds mRNA in the 70S ribosome, positioning it for translation
12	Fibrinogen-binding protein	cell surface-bound protein; virulence factor
13	Thioredoxin	reduction of other proteins by cysteine thiol-disulfide exchange

Table S3. Intracellular proteins with increased amount after growth in the presence of **F105** (LC-MS).

#	Protein	Function
1	3-hydroxyacyl-CoA dehydrogenase	oxidoreductase (involved in fatty acid metabolic processes), acts on the CH-OH group of donor
2	1-pyrroline-5-carboxylate dehydrogenase	oxidoreductase, acts on the CH-NH group of donors icipates in glutamate metabolism and arginine and proline metabolism)
3	Phosphoribosylamine--glycine ligase	purine biosynthesis
4	Glutamate dehydrogenase	glutamate to α-ketoglutarate, and vice versa
5	Ornithine--oxo-acid aminotransferase	Catalyzes the interconversion of ornithine to glutamate semialdehyde
6	Heme peroxidase	a number of oxidative reactions
7	Transaldolase	non-oxidative phase of the pentose phosphate pathway

Table S4. *S. aureus* proteins with altered mobility after incubation with **F105** *in vitro*.

#	Protein	Function
1	transketolase	pentose phosphate pathway
2	enolase	glycolysis
3	formate--tetrahydrofolate ligase	purine biosynthesis
4	thymidine phosphorylase	purine metabolism/pyrimidine metabolism
5	molecular chaperone GroEL	folding of many proteins
6	molecular chaperone DnaK	Folding
7	cysteine synthase	Biosynthesis of cysteine
8	inosine-5-monophosphate dehydrogenase	biosynthesis of guanine nucleotides
9	thioredoxin reductase	redox signaling,<br>reduction of other proteins by cysteine thiol-disulfide exchange
10	arginase	arginine + H_2O → ornithine + urea
11	CoA-disulfide reductase	oxidoreductases, specifically those acting on a sulfur group of donors with NAD+ or NADP+ as acceptor
12	branched-chain alpha-keto acid dehydrogenase subunit E2	catalyzes a transfer of the acyl group from the lipoyl moiety to coenzyme A (a stoichiometric cofactor)
13	glutaryl-CoA dehydrogenase	flavin adenine dinucleotide binding; oxidoreductase activity, acting on the CH-CH group of donors
14	succinyl-CoA synthetase subunit alpha	catalyzes the reversible reaction of succinyl-CoA to succinate
15	glyceraldehyde-3-phosphate dehydrogenase	glycolysis
16	serine hydroxymethyltransferase	simultaneous conversions of L-serine to glycine and tetrahydrofolate (THF)

References

Otsubo, T.; Minami, A.; Fujii, H.; Taguchi, R.; Takahashi, T.; Suzuki, T.; Teraoka, F.; Ikeda, K. 2-(Benzothiazol-2-yl)-phenyl-β-D-galactopyranoside derivatives as fluorescent pigment dyeing substrates and their application for the assay of β-D-galactosidase activities. *Bioorg. Med. Chem. Lett.* **2013**, *23*, 2245–2249.

Blazecka, P.G.; Belmont, D.; Curran, T.; Pflum, D.; Zhang, J. Further utilization of mucohalic acids: palladium-free, regioselective etherification and amination of α,β-dihalo γ-methoxycarbonyloxy and γ-acetoxy butenolides. *Org. Lett.* **2003**, *5*, 5015–5017.

Sheldrick, G.M. SHELXT – Integrated space-group and crystal-structure determination. *Acta Cryst.* **2015**, *A71*, 3–8.

Sheldrick, G.M. Crystal structure refinement with SHELXL. *Acta Cryst.* **2015**, *C71*, 3–8.

Farrugia, L.J. WinGX and ORTEP for Windows: an update. *J. Appl. Cryst.* **2012**, *45*, 849–854.
